# Genetic mutations linked to Parkinson's disease differentially control nucleolar activity in pre-symptomatic mouse models

**DOI:** 10.1242/dmm.028092

**Published:** 2017-05-01

**Authors:** Valentin Evsyukov, Andrii Domanskyi, Holger Bierhoff, Suzana Gispert, Rasem Mustafa, Falk Schlaudraff, Birgit Liss, Rosanna Parlato

**Affiliations:** 1 Institute of Anatomy and Medical Cell Biology, University of Heidelberg, 69120Heidelberg, Germany; 2 German Cancer Research Center, Molecular Biology of the Cell I, 69120Heidelberg, Germany; 3 Institute of Biotechnology, University of Helsinki, 00014 Helsinki, Finland; 4 German Cancer Research Center, Molecular Biology of the Cell II, 69120Heidelberg, Germany; 5 Department of Biochemistry, Institute of Biochemistry and Biophysics, Center for Molecular Biomedicine (CMB), Friedrich Schiller University Jena, 07743 Jena, Germany; 6 Leibniz-Institute on Aging – Fritz Lipmann Institute (FLI), 07743Jena, Germany; 7 Experimental Neurology, Goethe University Medical School, 60590Frankfurt am Main, Germany; 8 Institute of Applied Physiology, University of Ulm, 89081Ulm, Germany

**Keywords:** Nucleolus, Parkinson's disease, Neuronal homeostasis, Digenic model, rRNA

## Abstract

Genetic mutations underlying neurodegenerative disorders impair ribosomal DNA (rDNA) transcription suggesting that nucleolar dysfunction could be a novel pathomechanism in polyglutamine diseases and in certain forms of amyotrophic lateral sclerosis/frontotemporal dementia. Here, we investigated nucleolar activity in pre-symptomatic digenic models of Parkinson's disease (PD) that model the multifactorial aetiology of this disease. To this end, we analysed a novel mouse model mildly overexpressing mutant human α-synuclein (hA53T-SNCA) in a PTEN-induced kinase 1 (PINK1/PARK6) knockout background and mutant mice lacking both DJ-1 (also known as PARK7) and PINK1. We showed that overexpressed hA53T-SNCA localizes to the nucleolus. Moreover, these mutants show a progressive reduction of rDNA transcription linked to a reduced mouse lifespan. By contrast, rDNA transcription is preserved in DJ-1/PINK1 double knockout (DKO) mice. mRNA levels of the nucleolar transcription initiation factor 1A (*TIF-IA*, also known as *RRN3*) decrease in the substantia nigra of individuals with PD. Because loss of TIF-IA, as a tool to mimic nucleolar stress, increases oxidative stress and because DJ-1 and PINK1 mutations result in higher vulnerability to oxidative stress, we further explored the synergism between these PD-associated genes and impaired nucleolar function. By the conditional ablation of *TIF-IA*, we blocked ribosomal RNA (rRNA) synthesis in adult dopaminergic neurons in a DJ-1/PINK1 DKO background. However, the early phenotype of these triple knockout mice was similar to those mice exclusively lacking TIF-IA. These data sustain a model in which loss of DJ-1 and PINK1 does not impair nucleolar activity in a pre-symptomatic stage. This is the first study to analyse nucleolar function in digenic PD models. We can conclude that, at least in these models, the nucleolus is not as severely disrupted as previously shown in DA neurons from PD patients and neurotoxin-based PD mouse models. The results also show that the early increase in rDNA transcription and nucleolar integrity may represent specific homeostatic responses in these digenic pre-symptomatic PD models.

## Abstract (precis)

**Summary:** Genetic mutations linked to Parkinson's disease lead to stage-specific deregulation of the nucleolus, a major integrator of the cellular stress response.

## INTRODUCTION

The nucleolus is a controller of neuronal homeostasis and a central regulator of the cellular stress response (Boulon et al., 2010). Nucleolar activity is tightly linked to cellular well-being, as rRNA synthesis decreases in response to adverse extracellular conditions, including DNA damage, oxidative stress and neurotrophic withdrawal (Hetman and Pietrzak, 2012). Accordingly, reduced rDNA transcription and disruption of nucleolar integrity are common to several neurodegenerative disorders (Grummt, 2013; Parlato and Kreiner, 2013). Interestingly, mutant proteins and RNAs may directly interfere with the RNA polymerase I machinery, reducing the level of rDNA transcription in polyglutamine diseases, including Huntington's disease (HD) and in some forms of amyotrophic lateral sclerosis and fronto-temporal dementia (ALS/FTD) (Parlato and Bierhoff, 2015).

Impaired nucleolar activity and disrupted nucleolar integrity, known as nucleolar stress, have also been reported in Parkinson's disease (PD) autopsies in dopaminergic (DA) neurons (Rieker et al., 2011; Parlato and Liss, 2014). Notably, nucleolar stress is evident in neurotoxin-based PD mouse models upon analysis of rDNA synthesis and by the altered distribution of nucleolar proteins (Rieker et al., 2011; Healy-Stoffel et al., 2013). We have previously shown that induction of nucleolar stress in DA neurons leads to progressive and selective degeneration of substantia nigra (SN), impaired mitochondrial function and increased oxidative stress linked to downregulation of the mammalian target of rapamycin (mTOR) pathway (Rieker et al., 2011; Kreiner et al., 2013). The observation that neuronal death is accelerated in the acute neurotoxin-based *N*-methyl-4-phenyl-1,2,3,6-tetrahydropyridine (MPTP) model upon nucleolar stress revealed a mechanistic crosstalk between impaired nucleoli and mitochondria (Rieker et al., 2011).

Most PD cases are idiopathic, ∼10% of PD cases are Mendelian inherited and about 27% of heritability has been recently estimated (Mullin and Schapira, 2015). Several genetic mutations and risk factors for PD have been identified, revealing shared and converging pathophysiological pathways (Mullin and Schapira, 2015; Kumaran and Cookson, 2015; Duda et al., 2016). Interestingly, a PD-linked mutant form of the DJ-1 (*PARK7*) gene impaired maturation of rRNA in a cellular PD model upon proteasome inhibition (Vilotti et al., 2012). Conditional ablation of parkin (*PRKN* also known as PARK2) in DA neurons results in reduced level of precursor rRNA (pre-rRNA) transcripts and release of nucleolar proteins in the nucleus, a sign of nucleolar stress (Kang and Shin, 2015).

Despite the emerging evidence, the time- and cell-specific link between genetic mutations accounting for PD and nucleolar activity is still poorly investigated. To this end, mouse models based on PD genetic mutations represent a very convenient tool to dissect mechanisms underlying neuronal homeostasis in pre-symptomatic PD stages, as in general they are not affected by neuronal death (Dawson et al., 2010). DJ-1 and PINK1 (also known as PARK6) for example are known to protect against oxidative stress and to regulate mitochondrial function and clearance (Kim et al., 2005; Narendra et al., 2010). Mutations in these genes cause autosomal recessive early-onset PD; however compensatory mechanisms have been reported in the knockout mice, in which DA neuronal survival is not impaired, even in aged mutant mice (Pham et al., 2010; Glasl et al., 2012). This lack of DA neurodegeneration is also observed in transgenic mice overexpressing PD-related α-synuclein (*SNCA*) point mutations, sustaining the hypothesis that PD is a multifactorial neurodegenerative disorder (Dawson et al., 2010).

To focus the analysis on the integration of multiple pathways in PD, we performed a systematic analysis of rDNA transcription and nucleolar integrity in a novel digenic mouse model of PD. In this model, the heterologous prion promoter drives an exclusively neuronal overexpression (1.5-fold) of the PD-triggering human A53T point mutation in SNCA (hA53T-SNCA) (Gispert et al., 2003; Kurz et al., 2010). This mouse line was named hA53T-SNCA, and is also known as PrPmtA. In the double mutant mice, this mild overexpression of hA53T-SNCA as a neurotoxic stressor is complemented with a PTEN-induced kinase 1 knockout (PINK1 KO) background, leading to a loss of stress responses (hA53T-SNCA/PINK1KO) at different stages (Gispert et al., 2015; Auburger et al., 2016). hA53T-SNCA/PINK1KO mice showed a phenotype more severe than each respective single mutant, in particular, spontaneous activity at 3 months and lifespan were reduced, concomitantly with altered degradation and aggregation of SNCA protein at 18 months (Gispert et al., 2015). These characteristics were crucial when the mouse line was chosen for this study.

In addition, we studied adult mutant mice lacking DJ-1 and PINK1 (Pham et al., 2010; Glasl et al., 2012). DJ-1KO mice show ∼6% DA neuronal loss only in the ventral tegmental area independent of age and slight alterations in exploratory/motivational and cognitive behaviour (Pham et al., 2010). PINK1 KO mice show alterations in gait and olfactory function, but no alterations in DA neuron survival examined at 6 and 19 months (Glasl et al., 2012). Previous studies based on the triple knockout of parkin, DJ-1 and PINK1 have also shown no effects on DA neuronal survival, at least until 24 months (Kitada et al., 2009). These combined genetic models were therefore suitable to investigate the impact of PD-related genes on nucleolar function in pre-symptomatic models independently of DA neuronal loss.

We showed that the hA53T-SNCA mutation in a PINK1 KO background leads to reduced pre-rRNA levels and changes in number of nucleoli, accompanied by the progression towards a more severe phenotype. On the contrary, DJ-1 and PINK1 double knockout mice do not show reduced levels of rRNA synthesis. Because loss of DJ-1 and PINK1, and inhibition of rRNA synthesis alter mitochondrial function and render DA neurons more vulnerable to oxidative stress, we further investigated the functional link between these PARK genes and impaired nucleolar activity, which is known to increase oxidative stress (Rieker et al., 2011; Kreiner et al., 2013). We induced nucleolar stress in adult mice by the conditional genetic ablation of the RNA polymerase I regulator transcription initiation factor-1A (TIF-IA) in the DJ-1/PINK1 double knockout (DKO) background (Rieker et al., 2011). The phenotype of the TIF-IA single mutants was not enhanced in the triple knockout mice, further indicating that loss of DJ-1 and PINK1 does not regulate nucleolar function in these mouse models of pre-symptomatic PD.

Taken together, these results support the role of nucleolar-dependent mechanisms in the pre-symptomatic PD phase, and reveal differential effects of PARK mutations on nucleolar activity of SN DA neurons, as well as on homeostatic responses that target nucleolar function in preclinical stages.

## RESULTS

### Nucleolar activity is altered in a pre-symptomatic PD model based on mild overexpression of human A53T-SNCA in a *PINK1*-null genetic background

**Fig. 1.**
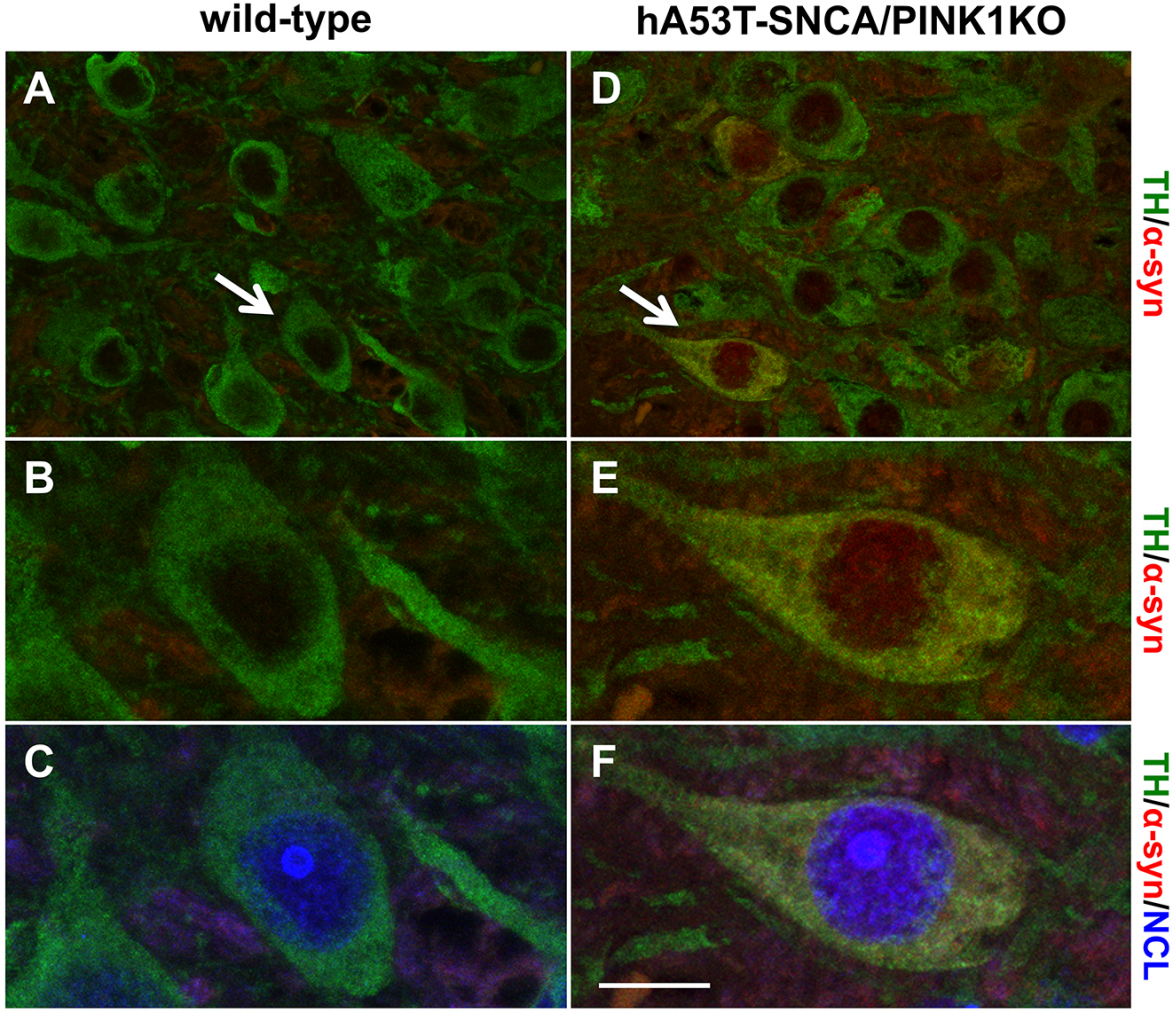
**hA53T-SNCA is expressed in DA neurons in the cytoplasm, the nucleus and the nucleolus.** Representative confocal microscopy images of paraffin sections immunostained with TH (green), human SNCA (α-syn; red)- and NCL (blue)-specific antibodies from 3-month-old wild-type (A-C) and hA53T-SNCA-overexpressing transgenic mice in a PINK1 KO background (hA53T-SNCA/PINK1KO) (D-F). B,C and E,F show higher magnifications of the region indicated by the arrows in A and D, respectively. Scale bar: 25 μm (A,D) and 12 µm (B-F).

In hA53T-SNCA/PINK1KO mice, behavioural abnormalities occur at 3 months, while increased protein aggregates are visible in the ventral midbrain between 15 and 17 months (Gispert et al., 2015) and the lifespan of a subset of these mice is reduced starting from 16 months (Gispert et al., 2015). In light of these phenotypes and because of the decreased viability after 16 months, we used 3-month-old mice as the early stage, and mice aged 16 and 19 months to represent the late stages in our analysis. By immunofluorescence staining with a specific human anti-SNCA antibody, we confirmed that hA53T-SNCA transgenic mice overexpress SNCA in the cytoplasm and nucleus of ventral midbrain DA neurons identified by tyrosine hydroxylase (TH) immunoreactivity at 3 months in comparison with the wild type (Fig. 1; Fig. S1) (Gispert et al., 2015). By the immunostaining with a nucleolin (NCL)-specific antibody, to identify the nucleolus, we showed that although hA53T-SNCA is completely absent in the wild-type DA neurons as expected (Fig. 1A-C), it is located in the nucleus and in the nucleolus in DA neurons of hA53T-SNCA/PINK1KO mice (Fig. 1D-F).

**Fig. 2.**
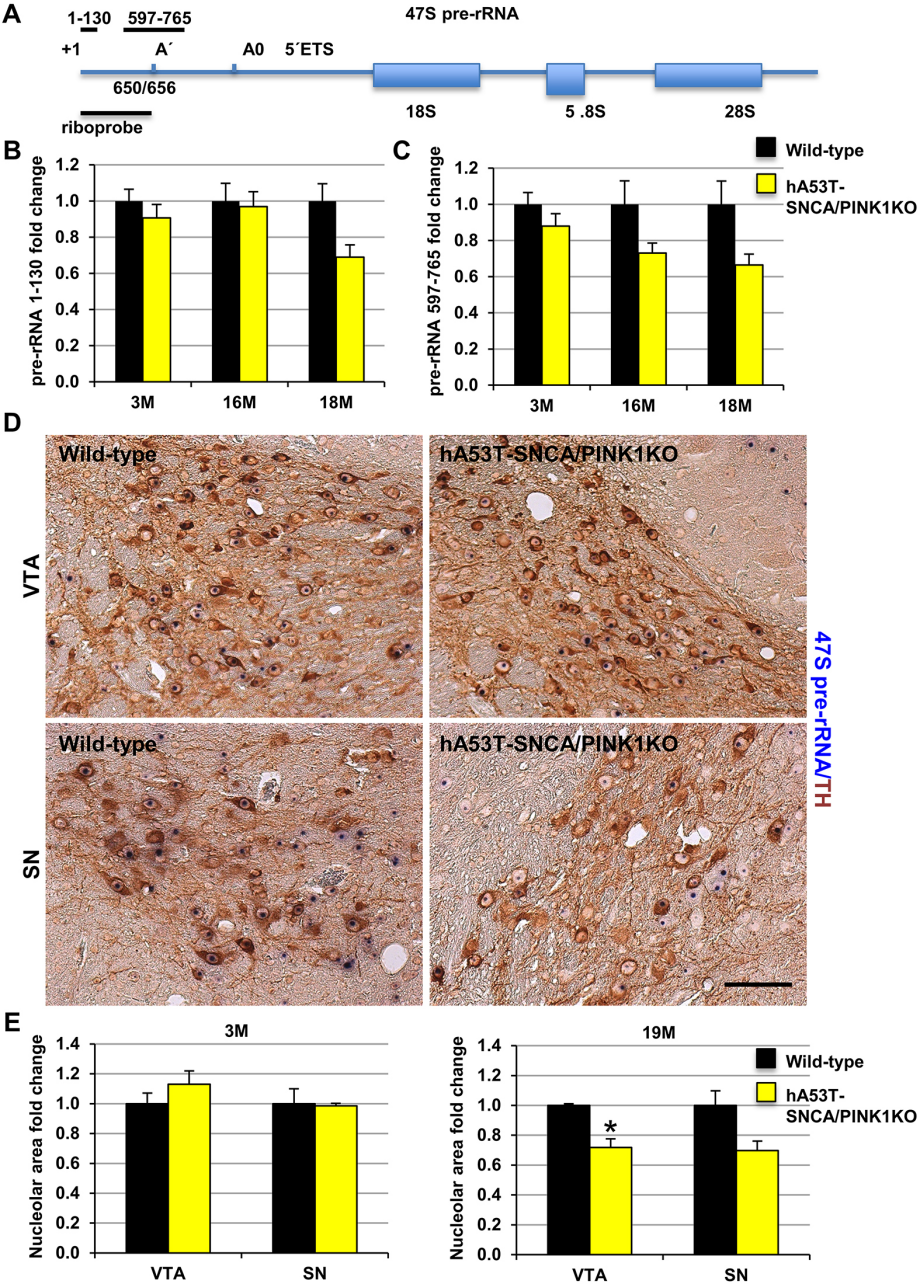
**rDNA transcription levels and reduced nucleolar area in DA neurons of hA53T-SNCA/PINK1KO mice at early symptomatic stages.** (A) Schematic representation of the 47S pre-rRNA transcript including the positions of the riboprobe used for ISH, primers used for qRT-PCR and the A′ and A0 cleavage sites within the 5′ external transcribed spacer (ETS). (B,C) Analysis of pre-rRNA (1-130) and pre-rRNA (597-765) by qRT-PCR at 3, 16 and 18 months in the ventral midbrain of wild-type (*N*=8,3,3) and hA53T-SNCA/PINK1KO mice (*N*=6,3,3) expressed as fold change to respective controls and normalized by *Gapdh*. (D) Representative images of ISH with 47S-specific riboprobe (blue) in combination with IHC by a TH-specific antibody (brown) at 19 months in VTA and SN of wild-type and hA53T-SNCA/PINK1KO mice. (E) Quantification of the area occupied by the 47S staining in hA53T-SNCA/PINK1KO expressed as fold change relative to respective controls in SN and VTA at 3 and 19 months (*N*=4 and 3 mice, respectively). Data are mean±s.e.m.; *P<0.05 in comparison to wild-type mice, as determined by Student's unpaired *t*-test. Scale bar: 70 μm.

Next, to monitor rDNA transcription, we detected 47S pre-rRNA (1-130) and another intermediate precursor (597-765) by quantitative real-time PCR (qRT-PCR) in dissected ventral midbrain at 3, 16 and 18 months in hA53T-SNCA/PINK1KO and respective age-matched controls (Fig. 2A-C). By this approach, the differences in pre-rRNA levels observed in hA53T-SNCA/PINK1KO mice at 18 months (Fig. 2B,C; Fig. S2A,B) in concomitance with a reduced lifespan and manifestation of the symptomatic phase, were not significant. Given the heterogeneous expression of the transgene across DA neurons and the different neuron populations, to provide a deeper insight of the rate of rRNA synthesis at the level of single DA cells, we performed *in situ* hybridization (ISH) in combination with immunohistochemistry (IHC) with anti-TH antibody and visualized full-length 47S pre-rRNA in DA neurons of SN and ventral tegmental area (VTA) in tissue sections (Fig. 2A,D,E). The stained area identifying the nucleolar pre-rRNA signal was about 30% lower in hA53T-SNCA/PINK1KO mice at 19 months of age in comparison to controls, suggesting a decreased amount of 47S pre-rRNA selectively in VTA DA neurons (Fig. 2E, right), but not SN DA neurons of hA53T-SNCA/PINK1KO mice (Fig. 2E, right; Fig. S2C,D).

The initial description of the hA53T-SNCA transgenic mice with exclusive neuronal expression driven by the prion promoter also showed human SNCA immunoreactivity in the hippocampus (Gispert et al., 2003). Accordingly, we also analysed 47S pre-rRNA signals at late stages in this region; however, the differences between the hA53T-SNCA/PINK1KO mutant and respective control mice, were not significant (Fig. S3).

**Fig. 3.**
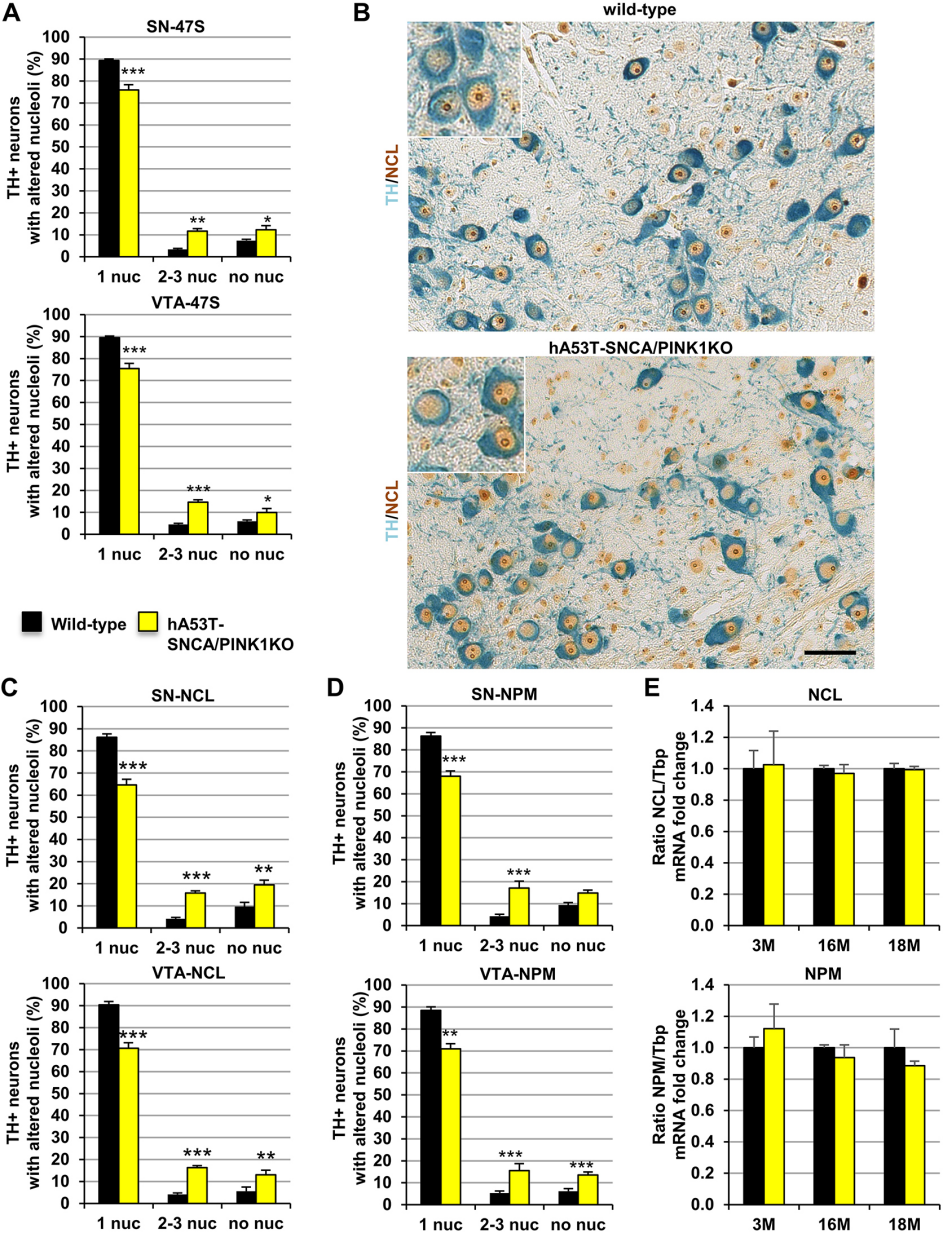
**Nucleolar number is altered in hA53T-SNCA/PINK1KO mice at pre-symptomatic stages.** (A) Quantification of nucleoli detected by ISH with 47S-specific riboprobe in TH-positive neurons of 3-month-old wild-type and hA53T-SNCA/PINK1KO mice (*N*=4) in SN (left) and VTA (right) expressed as percentage of TH-positive neurons with 1, 2-3 or no nucleoli (nuc). (B) Representative images of ventral midbrain paraffin sections from 3-month-old wild-type and hA53T-SNCA/PINK1KO mice immunostained with NCL (brown)- and TH (blue)-specific antibodies. (C,D) Quantification of the number of nucleoli detected by NCL (C) and NPM (D) immunostaining in TH-positive neurons of wild-type and hA53T-SNCA/PINK1KO mice in SN (top) and VTA (bottom) expressed as percentage of TH-positive neurons with 1, 2-3 or no nucleoli (*N*=4 mice). (E) Analysis of *Ncl* (top) and *Npm* (bottom) mRNA levels by qRT-PCR at 3, 16 and 18 months in wild-type (*N*=5,3,3) and hA53T-SNCA/PINK1KO mice (*N*=6,3,3) expressed as fold change to respective controls and normalized by *Tbp*. All data are mean±s.e.m.; *P<0.05; **P<0.01; ***P<0.001 in comparison to wild-type, as determined by two-way ANOVA followed by Sidak's multiple comparison test. Scale bar: 50 μm.

A similar analysis performed at 3 months in DA neurons of hA53T-SNCA/PINK1KO mice did not reveal any significant changes of pre-rRNA (Fig. 2E, left). Interestingly, a quantitative analysis of 47S pre-rRNA signals in DA neurons at 3 months showed that the percentage of TH-positive neurons containing one nucleolus was lower in SN and VTA of mutant mice (Fig. 3A; Fig. S4A,B). Concomitantly, the percentage of DA neurons showing no nucleolar staining increased (Fig. 3A). Nevertheless, the percent of TH-positive neurons with more than one 47S signal was also increased in the hA53T-SNCA/PINK1KO mutant mice, suggesting that in a subset of DA neurons transcription of rDNA was promoted (Fig. 3A). These data were supported by IHC with NCL- and nucleophosmin/B23 (NPM)-specific antibodies in combination with TH immunostaining (Fig. 3B-D). Analysis of the distribution of these two independent nucleolar markers in TH-positive neurons showed that the number of neurons containing two or three nucleoli was significantly higher in the hA53T-SNCA/PINK1KO mutant mice (Fig. 3C,D; Fig. S4C-F). This pattern of staining might suggest the activation of compensatory mechanisms promoting nucleolar activity in young mice.

To further explore the hypothesis that reduced levels of 47S pre-rRNA and altered nucleolar integrity at late stages could be linked to reduced expression of rRNA synthesis regulators, we used qRT-PCR to analyse the levels of *Ncl* and *Npm1* (*B23*) mRNA, which encode nucleolar proteins known to play a role in the regulation of rRNA synthesis, in the ventral midbrain samples from hA53T-SNCA/PINK1KO mice and age-matched controls at 3, 16 and 18 months (Fig. 3E and Fig. S4G,H). No differences were detected, suggesting that impaired nucleolar activity detected by qRT-PCR and by ISH was not linked to a general transcriptional deficit in the ventral midbrain.

**Fig. 4.**
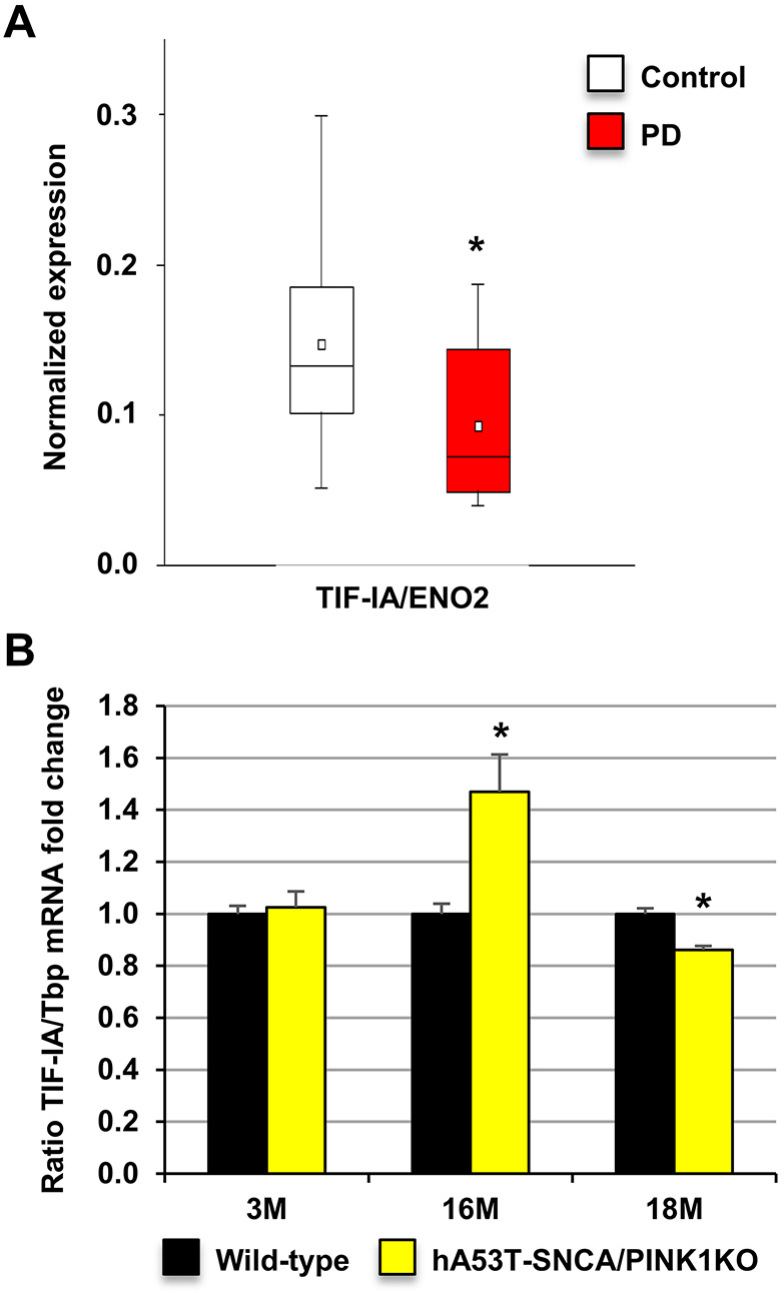
**Expression of TIF-IA that regulates rRNA synthesis in PD patients and hA53T-SNCA/PINK1KO mice.** (A) TIF-IA expression in ventral midbrain DA neurons in brain autopsies from PD patients (*N*=5, *n*=20 sections) and age-matched controls (*N*=8, *n*=16 sections) expressed as fold change compared with controls normalized by ENO2; *P=0.0192, as determined by Wilcoxon rank sum test. Data are mean±s.e.m. (B) Analysis of *TIF-IA* expression by qRT-PCR in 3-, 16- and 18-month-old wild-type (*N*=8,3,3) and hA53T-SNCA/PINK1KO mice (*N*=6,3,3) expressed as fold change to respective controls normalized by *Tbp*. Data are mean±s.e.m.; *P<0.05 in comparison to wild-type mice, as determined by Student's unpaired *t*-test.

Interestingly, *TIF-IA* mRNA levels were significantly lower in the ventral midbrain of human post-mortem PD brain samples compared with those from age-matched controls (control, *N*=8 and PD, *N*=5; Fig. 4A). These findings support previous evidence of impaired rRNA synthesis in PD symptomatic stages (Kang and Shin, 2015; Rieker et al., 2011); however, they need to be interpreted with caution given the different cellular composition of the human ventral midbrain tissue in control and PD patients. To further investigate a link between PD-related mutations and nucleolar activity in hA53T-SNCA/PINK1KO models, we analysed the expression of TIF-IA in hA53T-SNCA/PINK1KO mice. Similar to the human PD samples, *TIF-IA* mRNA in mouse ventral midbrain tissue was reduced at a clearly symptomatic stage (18 months) after a surprising transitory upregulation phase at 16 months (Fig. 4B; Fig. S5).

### rDNA transcription is unaltered in a pre-symptomatic PD model based on the genetic inactivation of DJ-1 and PINK1

**Fig. 5.**
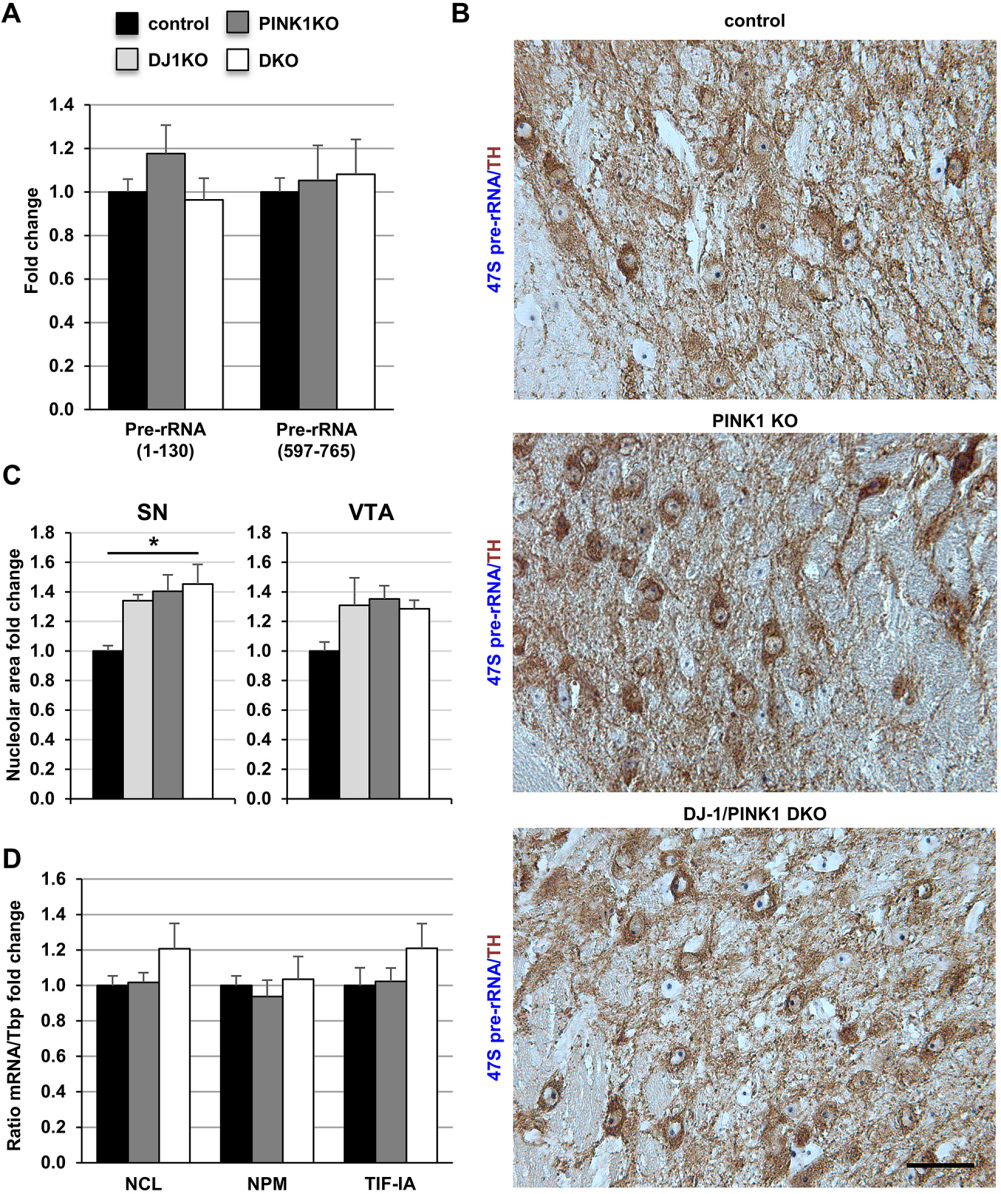
**rDNA transcription is not reduced in PINK1 KO or DJ-1/PINK1 DKO mice.** (A) Analysis of pre-rRNA (1-130) and pre-rRNA (597-765) by qRT-PCR in controls (*N*=3), PINK1 KO (*N*=8) and DJ-1/PINK1 DKO mice (*N*=8) expressed as fold change to respective controls normalized by *Gapdh*. (B) Representative images of ISH with a 47S-specific riboprobe (blue) in combination with immunohistochemistry with a TH-specific antibody (brown) in 9-month-old control, PINK1 KO and DKO mice. (C) Quantification of the area occupied by the 47S staining in control, DJ-1KO, PINK1 KO and DKO mice expressed as fold change relative to respective controls in SN and VTA (*N*=3 mice). (D) Analysis of *Ncl*, *Npm* and *TIF-IA* mRNA levels by qRT-PCR in wild-type (*N*=3), PINK1 KO (*N*=8) and DJ-1/PINK1 DKO (*N*=8) mice at 9 months expressed as fold change to respective controls normalized by *Tbp*. All data are mean±s.e.m. *P<0.05 as determined by one-way ANOVA followed by Tukey's multiple comparisons test. Scale bar: 50 μm.

Similarly, we analysed nucleolar activity in genetic models of pre-symptomatic PD based on autosomal recessive PARK gene mutations. Parkin mutant mice have been previously analysed (Kang and Shin, 2015). Here, we focused on DJ-1/PINK1 DKO mice and control littermates. By qRT-PCR analysis, levels of pre-rRNA (1-130) and the intermediate precursor (597-765) were similar in dissected ventral midbrain samples from adult control *Pink1*^−/−^ and in DKO mice (7 months old) (Fig. 5A; Fig. S6A).

The quantitative analysis of 47S pre-rRNA signal in DA neurons after ISH in combination with TH immunostaining did not reveal a decreased stained area in TH-positive neurons of the individual single knockouts or DKO mice, further supporting the conclusion that the loss of both DJ-1 and PINK1 did not impair rDNA transcription (Fig. 5B,C). On the contrary, the stained area identifying the nucleolar 47S signal in SN DA neurons of DKO mice, was significantly larger than that of controls, while the other differences did not reach statistical significance (Fig. 5C; Fig. S6B). The analysis of *Ncl*, *Npm* and *TIF-IA mRNA* levels in ventral midbrain from control, PINK1 KO and DJ-1/PINK1 DKO mice did not show any significant difference (Fig. 5D; Fig. S6C). These results indicate that pre-symptomatic PD models activate compensatory transcriptional mechanisms maintaining RNA polymerase I activity; however, symptomatic stages are, in general, associated with disrupted nucleolar function and integrity (Rieker et al., 2011).

### Early PD-like phenotypes caused by loss of TIF-IA are not enhanced by DJ-1/PINK1-dependent networks

These results indicated that unlike results seen with hA53T-SNCA/PINK1KO and previously reported neurotoxin-based models (Healy-Stoffel et al., 2013; Rieker et al., 2011), loss of DJ-1 and PINK1 did not impair nucleolar activity in DA neurons.

The observation that DA-specific TIF-IA conditional KO mice (cKO) are more vulnerable to acute MPTP treatment suggested the interaction of nucleolar- and mitochondrial-dependent pathways in these models of neurodegeneration (Rieker et al., 2011). Similarly, because both DJ-1 and PINK1 play a neuroprotective role against oxidative stress, we tested the hypothesis that DJ-1/PINK1 KO could exacerbate the effects of nucleolar stress in DA neurons, suggesting that the pathways regulated by DJ-1 and PINK1 interact with those triggered by nucleolar stress.

**Fig. 6.**
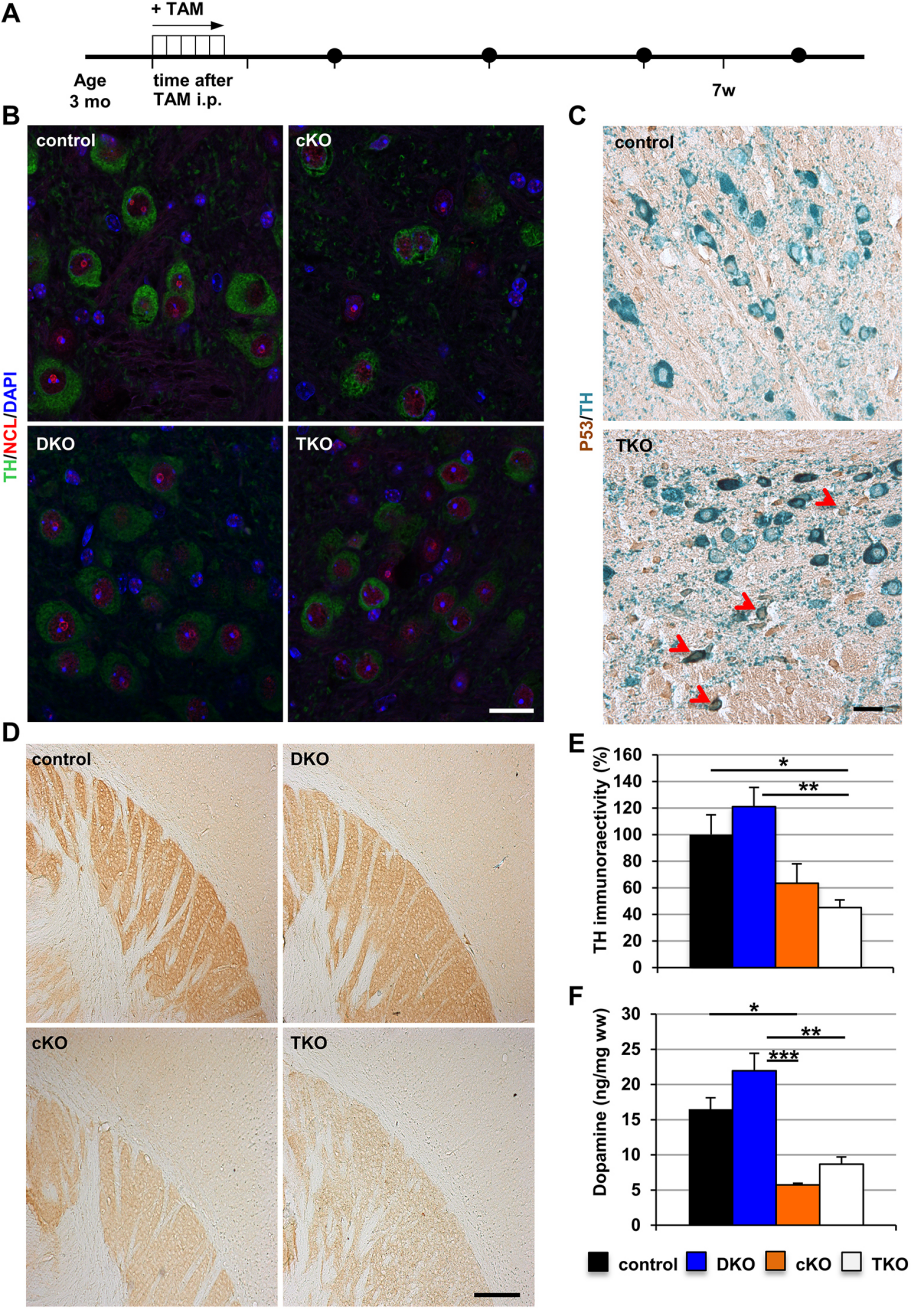
**Loss of DJ-1 and PINK1 does not exacerbate the toxic effects of nucleolar stress in DA neurons.** (A) Experimental design. (B) Representative confocal images showing nucleolar integrity in DA neurons by immunofluorescence staining with NCL (red) and TH (green) antibodies in control, DJ-1/PINK1 DKO, TIF-IA conditional mutants (cKO) and TKO mice. (C) Representative IHC images of p53 protein (brown) in TH-positive neurons (blue) in control and TKO mice 7 weeks after TAM injections. Neurons showing increased p53 levels are indicated by arrowheads. (D) Representative images of striatal TH immunoreactivity on vibratome sections of control, DJ-1/PINK1 DKO, TIF-IA conditional mutants (cKO) and TKO mice. (E) Quantification of TH immunoreactivity in the striata of control (*N*=5), DKO (*N*=3), cKO (*N*=4) and TKO (*N*=5) mice. (F) Analysis of dopamine content by HPLC-ED in the striata of control (*N*=13), DKO (*N*=9), cKO (*N*=4) and TKO (*N*=5) mice. Data are mean±s.e.m.; *P<0.05; **P<0.01; ***P<0.001 as determined by one-way ANOVA followed by Tukey's multiple comparison test. ww, wet weight; TAM, tamoxifen. Scale bar: 30 μm (B), 50 μm (C) and 200 μm (D).

To this end, we generated inducible DA TIF-IA cKO mice lacking DJ-1 and PINK1 (triple KO, TKO) and analysed mice at 3 months and 7 weeks after tamoxifen injection, a stage at which no significant neuronal death has yet occurred in the DA TIF-IA cKO mice (Rieker et al., 2011) (Fig. 6A). First, we confirmed that nucleolar integrity was disrupted in DA neurons of TIF-IA cKO and TKO by confocal analysis of immunofluorescence staining with NCL- and TH-specific antibodies (Fig. 6B). Similarly, we showed that NCL distribution was unaltered in the DJ-1/PINK1 DKO mice. Moreover, the induction of nucleolar stress in DA neurons was confirmed by IHC showing increased p53 protein levels in TKO mice (Fig. 6C), as p53 levels are known to increase as a result of nucleolar stress (Rieker et al., 2011).

However, TH immunoreactivity in the striatum of cKO and TKO mice was comparable, indicating that loss of DJ-1 and PINK1 does not exacerbate the toxic effects of nucleolar stress (Fig. 6D,E; Fig. S7A). These results were supported by the comparable levels of dopamine in the striata of TIF-IA cKO and TKO mice measured by high-performance liquid chromatography followed by electrochemical detection (HPLC-ED) (Fig. 6F; Fig. S7B). In summary, the results for the DKO and TKO indicate that loss of DJ-1 and PINK1 does not impair nucleolar activity and integrity in a pre-symptomatic stage, in stark contrast to the hA53T-SNCA/PINK1KO model.

## DISCUSSION

Nucleolar stress is emerging as one important factor in neurodegenerative diseases (Hetman and Pietrzak, 2012; Parlato and Bierhoff, 2015). In general, for as yet unclear reasons, inhibition of rRNA synthesis could be both neuroprotective and neurotoxic (Kreiner et al., 2013). Our study shows a differential effect of PD-related (PARK) genetic mutations on nucleolar activity. While hA53T-SNCA/PINK1KO did affect nucleolar activity, knockout of DJ1 (PARK7) and PINK1 (PARK6) did not. Our results also show that nucleolar activity decreases with increased phenotypic severity, reinforcing its role as a stress sensor. The localization of mutant SNCA in the nucleoli of DA neurons of hA53T-SNCA/PINK1KO mice suggests that this mutant protein may directly interfere with regulators of the RNA polymerase I transcriptional machinery. Because NCL is mainly localized in the dense fibrillar compartment where transcription and processing of rDNA take place, these steps of rRNA biogenesis might be altered in the hA53T-SNCA/PINK1KO mice (Sirri et al., 2008; Berger et al., 2015). A similar function is common to causative mutant proteins, for example, in polyglutamine diseases (Parlato and Bierhoff, 2015; Tsoi et al., 2012). However, based on this study, we cannot rule out the possibility that the hA53T-SNCA mutation is responsible for the altered nucleolar activity and integrity observed at 3 and 20 months. Given the observation that the PINK1 KO mice do not show the same phenotype as the hA53T-SNCA/PINK1KO double mutants, the hA53T-SNCA mutation might account for the decreased levels of pre-rRNA and reduced nucleolar area observed in the early symptomatic stage (from 16 months on).

The major advantage of the models examined here is that they do not show neurodegeneration, enabling us to dissect the impact of specific PD mutations on nucleolar activity and integrity independent of neuronal loss. Moreover, the experimental approaches adopted here allowed us to characterize rRNA synthesis in specific DA neurons at pre-symptomatic PD stages.

The altered distribution of nucleolar proteins such as NCL and NPM in hA53T-SNCA/PINK1KO mice confirms that nucleolar activity is downregulated and nucleolar integrity is disrupted at an early stage in a subset of DA neurons. Intriguingly, in the meantime, another group of DA neurons upregulate rRNA synthesis, as shown by the increased number of nucleoli, supporting the hypothesis that initial compensatory mechanisms target nucleolar activity. A similar condition has been also reported in motor neurons in a murine model of ALS in response to the disturbance of endoplasmic reticulum proteostasis and in response to proteasome inhibition in sensory ganglion neurons (Palanca et al., 2014; Riancho et al., 2014). In this regard, we should mention that the hypertrophy of cortical neurons and their nuclei and nucleoli in asymptomatic Alzheimer's disease may represent an early reaction to the presence of neurotoxic Aβ or tau, or a compensatory mechanism that prevents the progression of the disease into dementia (Iacono et al., 2008; Riudavets et al., 2007). This is linked to local Aβ-induced metabolic insults and neuronal death (Cohen et al., 2009). Our previous results show that the neuroprotective deletion of Pten in adult DA neurons also results in the increase of neuronal soma and nucleolar size (Domanskyi et al., 2011). Future studies are necessary to establish whether enhanced nucleolar activity at a pre-symptomatic stage is neuroprotective or on the other hand might even actively contribute to neurodegenerative pathways – with potential implications for design of therapeutic strategies. The next fundamental question is in fact to what extent a controlled downregulation of nucleolar function could even be beneficial under cellular stress (Mayer and Grummt, 2005). Hence, further studies are required to establish the role of the significant decrease of 47S pre-rRNA observed in the hA53T-SNCA/PINK1KO mice in VTA DA neurons. This observation suggests a differential regulation of nucleolar activity in more-resistant VTA DA neurons and highly vulnerable SN DA neurons, which are the most exposed DA population in PD, in line with the functional and metabolic differences between VTA and SN neurons (Duda et al., 2016). Of course, this differential vulnerability may simply be due to the heterologous prion promoter driving differing overexpression levels of hA53T-SNCA in the two neuronal populations. However, we cannot link this reduced nucleolar activity to a reduced neuronal survival in this model given its reduced lifespan. Ultimately the decreased pre-rRNA synthesis observed at the late stage in this model could be linked to ageing and/or be a consequence of the severe synaptic deficits in these mice (Johnson et al., 1998; Burke, 2010).

Future studies in other mouse models generated by a knock-in approach and expressing different point mutations in the human *SNCA* gene, such as A30P, that lead to familial PD forms, could provide further help to define in detail the link between specific PD mutations and nucleolar activity (Plaas et al., 2008; Antony et al., 2011).

Unlike dominant negative mutant forms of SNCA, DJ-1 and PINK1 loss-of-function mutations do not impair rDNA transcription in DA neurons. However, the DJ-1 L166P mutation that results in misfolded DJ-1 proteins with gain-of function effects, leads to an increase of rDNA transcription in SH-SY5Y cells upon proteasome inhibition unlike the DJ-1 loss-of-function mutation, which has no effect (Vilotti et al., 2012). Loss of DJ-1 has been shown to induce fragmentation of mitochondria and to cause a deficit in mitochondrial fusion, resulting in increased endogenous oxidative stress (Pham et al., 2010). Similarly, loss of PINK1 influences cellular sensitivity to toxins, protecting against mitochondrial fragmentation (Glasl et al., 2012). *Drosophila* Pink1 mutants show global downregulation of translation, suggesting that such cellular response compensates mitochondrial dysfunction by limiting energy consumption. In fact enhanced translation through S6 kinase activation significantly exacerbated Pink1 mutant phenotypes, whereas reduction of translation suppressed the phenotype (Liu and Lu, 2010). Based on the observation that loss of TIF-IA results in downregulation of mTOR activity, impaired mitochondrial activity and increased oxidative stress (Rieker et al., 2011; Kreiner et al., 2013), we expected an exacerbated impact of nucleolar stress in DA neurons lacking DJ-1/PINK1, in particular on DA striatal projections and dopamine content. However, the results of this study may indicate that DJ-1 and PINK1 function either upstream or independently of the rRNA control pathway, or that they trigger protective compensatory processes that lead to normal nucleolar function at pre-symptomatic stages.

The current link between PD-related mutations and rDNA synthesis regulators, such as NCL, NPM and TIF-IA is further summarized in Table 1. The expression levels of NCL are dramatically reduced in the SN of human PD subjects, compared with controls (Caudle et al., 2009) as well as with those of TIF-IA, and could account for the reduced rRNA synthesis in PD. NCL and also NPM may play a role in rRNA synthesis and in neuronal survival (Pfister and D'Mello, 2015), prompting us to also assume an important role of nucleolin in PD by regulation of rRNA synthesis. Evidence of reduced NPM in PD has been also recently provided (Garcia-Esparcia et al., 2015). Interestingly, both DJ-1 and SNCA associate with NCL; hence, an imbalance in this interaction could also alter rRNA synthesis (Jin et al., 2007). We could not detect any change in *Ncl* mRNA in any of the genetic pre-symptomatic PD mouse models; however, NCL and SNCA colocalize in hA53T-SNCA/PINK1KO, suggesting that NCL could be responsible for the observed changes in rRNA synthesis in these mutants through a yet to be elucidated mechanism. Further studies should address whether these mechanisms targeting the nucleolus occur in pre-symptomatic PD and in other genetic PD models. Mostly, it will be important to establish whether and when changes of nucleolar activity turn out to be fatal, what triggers them and why they have a cell-specific impact.

**Table 1.**
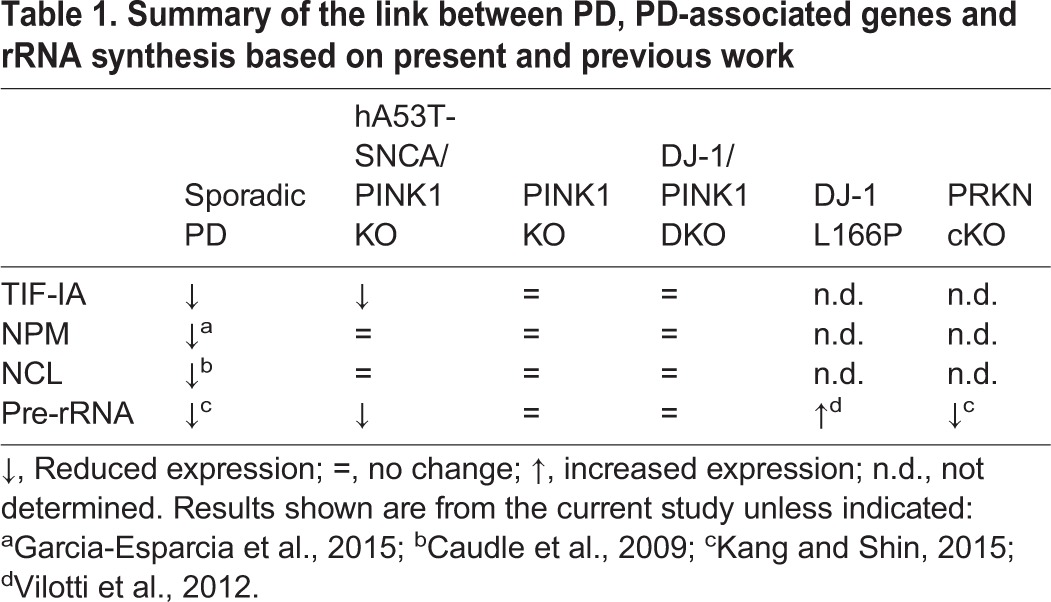
**Summary of the link between PD, PD-associated genes and rRNA synthesis based on present and previous work**

Conditional loss of parkin in DA neurons has been shown to downregulate rRNA synthesis (Kang and Shin, 2015). NPM and NCL interact with PARIS (parkin interacting substrate, ZNF746) and PARIS colocalizes with parkin in the nucleolus (Kang and Shin, 2015). Interestingly, overexpression of PARIS inhibits rRNA synthesis resulting in ∼30% less pre-rRNA 3 months after induction of the mutation. The same study shows that PARIS protein levels increases also in PD patients and that 47S pre-rRNA is reduced in the SN of PD patients.

There is growing evidence that DJ-1, PINK1 and parkin interact with each other, in particular PINK1 phosphorylates and activates parkin and DJ-1 physically interacts with the transcription factor forkhead box O3A (FOXO3A), to activate the *Pink1* promoter in DJ-1 KO mouse embryonic fibroblasts (Requejo-Aguilar et al., 2015). However, unlike parkin cKO mice, which show neurodegeneration at later stages, and also unlike PD- and neurotoxin-based models (Rieker et al., 2011; Garcia-Esparcia et al., 2015), RNA polymerase I activity is not severely impaired in hA53T-SNCA/PINK1KO, PINK1 KO or DJ-1/PINK1 DKO mice, supporting the hypothesis that compensatory responses might sustain nucleolar activity (Mullin and Schapira, 2015). Understanding these complex mechanisms is a prerequisite for novel neuroprotective strategies that are based on selective modulation of nucleolar function.

## MATERIALS AND METHODS

### Ethical use of human material and animals

Procedures involving animal care were approved by the Committee on Animal Care and Use (Regierungspräsidium Karlsruhe, 35-9185.81/G- 180/08) in accordance with the local Animal Welfare Act and the European Communities Council Directives (2010/63/EU and 2012/707/EU). Human post-mortem midbrain tissue blocks were provided by the German BrainNet, grant no. GA 28).

### Human samples

Detailed information on the 13 human post-mortem ventral midbrain tissues from controls and PD brains can be found in Schlaudraff et al. (2014). Mean age of controls (*N*=8) is 69±1.6 years and PD (*N*=5) is 78.2±1.3 years (Braak stages 0, II, III, IV, V).

### Mice

*Dj1*^−/−^ and *Pink1*^−/−^ mice in a C57Bl6/J background were generously donated by Dr Wolfgang Wurst (Helmholtz Zentrum München, Neuherberg, Germany) (Pham et al., 2010; Glasl et al., 2012). Homozygous *TIF-IA^flox/flox^; DATCreERT2* mutant mice were generated by crossing mice carrying the *TIF-IA* floxed allele to the transgenic line DATCreERT2. *TIF-IA^+/flox^; DATCreERT2*-positive mice were crossed again with *TIF-IA^flox/flox^* mice. The analysis of the genotype was performed as previously described (Rieker et al., 2011). These mice were crossed to DJ-1 and PINK1 double knockout mice to obtain triple knockout mice that lack TIF-IA in DA neurons after tamoxifen induction of CreERT2. To induce *TIF-IA* gene deletion in DA neurons, 2-month-old mice were injected intraperitoneally with 1 mg tamoxifen (TAM) twice a day for five consecutive days and were analysed 7 weeks after the last injection (Rieker et al., 2011). As controls, littermates with wild-type allele for all genes also injected with tamoxifen were examined. Both male and female mice were used for the experiments. PrPmtA mice overexpressing the human A53T-SNCA mutation were crossed with *Pink1*^−/−^ mice, as previously described (Gispert et al., 2015). For the experiments reported here, male and female mice were used.

### Histological analysis

Mice were sacrificed by cervical dislocation and brains were immediately dissected. For immunohistochemistry, one brain hemisphere was fixed in 4% paraformaldehyde overnight and paraffin embedded or sectioned on a vibratome (50 µm thickness); paraffin-embedded samples were sectioned to 7 µm thick. Vibratome sections containing the striatum comprised between Bregma +0.14 mm and −0.98 mm and paraffin sections from the midbrain region comprised between Bregma −2.54 mm and −3.80 mm were used for the analysis and incubated with primary antibodies overnight at 4°C. Visualization of antigen-bound primary antibodies following antigen retrieval (HK086-9K, Biogenex) was carried out using a biotinylated secondary antibody together with the avidin-biotin system and the Vector peroxidase kit (PK-6100, Vector Laboratories) using both diaminobenzidine tablets (D4293, Sigma) and HistoGreen HRP-substrate kit (E109, Vector) as a substrate. For immunofluorescence, Alexa 488 (A-21206), Alexa 594 (A-21207) and Alexa 647 (A-21448) (1:100, Thermo Fisher Scientific) secondary antibodies were used. Primary antibodies for immunostaining were: anti-α-synuclein (1:500, ab27766, Abcam), anti-nucleolin (1:500, ab70493, Abcam), anti-nucleophosmin (1:100, MAB4500, NPM/B23, Millipore), anti-tyrosine hydroxylase (1:500, AB1542, Millipore), anti-p53 (1:400, NCL-p53-CM5p, Novocastra).

Non-radioactive *in situ* hybridization (ISH) was performed on paraffin sections using a specific riboprobe hybridizing to regions in the leader sequence of the pre-rRNA followed by IHC with TH antibody as previously described (Rieker et al., 2011). The ISH signal was quantified by labelling the riboprobe with digoxigenin. This produced a defined signal area (approximately the size of nucleoli) that was used for quantification of the nucleolar area by ImageJ software (Rieker et al., 2011). For confocal analysis, images were acquired using a Leica SP8 system.

### RNA isolation and qRT-PCR

Total RNA was isolated from dissected mouse ventral midbrain in the region comprised between Bregma −2.54 mm and −3.80 mm. Levels of *Ncl*, *Npm*, *TIF-IA* mRNA and pre-rRNA were monitored by reverse transcription followed by quantitative real-time PCR (qRT-PCR). Synthesis of cDNA with M-MLV reverse transcriptase (SuperScript III First Strand Synthesis Supermix) (18080-400, Thermo Scientific) was primed with random hexamers. For detection of pre-rRNA, either the first 130 nucleotides relative to the transcription start site were amplified using the 5′-ACTGACACGCTGTCCTTTCC and 5′-GACAGCTTCAGGCACCGCGA primers or a primer pair covering the first processing site was used: 5′-CGTGTAAGACATTCCTATCTCG and 5′- GCCCGCTGGCAGAACGAGAAG. To amplify *Gapdh*, we used the following primers: 5′-CATGGCCTTCCGTGTTCCTA and 5′-GCGGCACGTCAGATCCA. pre-RNA levels relative to *Gapdh* mRNA levels were determined using SYBR Green chemistry (SYBR Green Mix, 04887352001, Roche) by a Light Cycler 480 instrument (Roche), as previously described (Kiryk et al., 2013). For each amplicon, serial dilutions of cDNA from the ventral midbrain were included in each run to generate standard curves for relative quantification by the Light Cycler 480 software. The relative changes in pre-rRNA expression were normalized to *Gapdh*, as a reference gene, after checking its stable expression. The analysis of the mouse ventral midbrain cDNA was performed by a StepOnePlus instrument (Applied Biosystems). The following TaqMan gene expression assays were used: TIF-IA (Mm01344420_m1), nucleolin (Mm01290591_m1), nucleophosmin (Mm02391781_g1), and Tbp (TATA-binding protein) (Mm00446973-m1) (Applied Biosystems/Life Technologies). For the TaqMan assays, the ΔΔCT method was used to normalize the mouse qRT-PCR data by the 2^−ΔΔCT^ formula (Livak and Schmittgen, 2001). The normalization was performed using the stably expressed reference gene *Tbp*. Expression changes were calculated as a fold change versus mean of respective control samples.

Total RNA from human post-mortem horizontal midbrain tissue cryosections was analysed on an Applied Biosystems GeneAmp 7900HT PCR instrument, as previously described (Gründemann et al., 2008, 2011; Schlaudraff et al., 2014). The human TIF-IA (Hs00255800_m1) and the ENO2 (Hs00157360_m1) TaqMan assays (Applied Biosystems) were used for real-time qPCR, and expression levels were calculated using respective standard curve data by the formula *S*^[(Ct-Yintercept)/slope]^, where *S* is the serial dilution factor of the standard curve. The data were calculated in respect to *ENO2* (neuron specific enolase), by using slopes of respective standard curves over 4 magnitudes as described in detail in (Schlaudraff et al., 2014). We have empirically proven that *ENO2* mRNA is expressed at constant levels in individual SN DA neurons (Liss et al., 2001).

### HPLC-ED

The striatum between Bregma +0.14 mm and −0.98 mm was dissected using a mouse brain matrix and dopamine content was measured per mg of wet weight (ww) by HPLC-ED, as previously described (Rieker et al., 2011).

### Statistical analysis

Eight coronal paraffin sections (every fourth section) per mouse were analysed within the ventral midbrain and hippocampus for ISH and IHC. ImageJ software was used to measure the area occupied by the 47S signal for at least 150 nucleoli per mouse. For DA neurons, the total area of the nucleolar signal per mouse was normalized to the number of TH-positive neurons in either SN or VTA (at least 150 cells per mouse in SN and at least 150 cells per mouse in VTA), in this way we obtained the average 47S signal area per cell per mouse. The average area per mouse from at least three mice per genotype at each age was used to calculate the average nucleolar area per genotype and age. The average area per genotype was then plotted as fold change relative to the average area of the respective control and the results are expressed as the mean of the fold changes ±s.e.m. Individual data plots are included in Figs S2,S4-S7. ImageJ was also used to measure TH immunoreactivity in the striatum in vibratome sections (Rieker et al., 2011). All measurements were carried out at least in triplicate as specified in the figure legends. Statistical significance of the results was analysed by one-way or two-way ANOVA, followed by *post hoc* tests for comparisons between multiple groups or by Student's *t*-test for comparisons between two groups of values, as indicated in figure legends, using GraphPad Prism (GraphPad) or SPSS Statistics (IBM) software packages. In all cases, *P*<0.05 was considered significant.
